# Worrying increase in the risk of vertical transmission of syphilis in Croatia, 2020 to 2024

**DOI:** 10.2807/1560-7917.ES.2024.29.36.2400517

**Published:** 2024-09-05

**Authors:** Tatjana Nemeth Blažić, Nina Krajcar, Mirjana Lana Kosanović Ličina, Dominik Ljubas, Otilia Mardh, Ivana Božičević

**Affiliations:** 1 Croatian Institute of Public Health, Zagreb, Croatia; 2 University Hospital for Infectious Diseases ‘Dr Fran Mihaljević’, Zagreb, Croatia; 3 Andrija Štampar Teaching Institute of Public Health, Zagreb, Croatia; 4 European Centre for Disease Prevention and Control (ECDC), Stockholm, Sweden; 5 World Health Organization Collaborating Centre for HIV Strategic Information, University of Zagreb School of Medicine, Zagreb, Croatia

**Keywords:** congenital syphilis, pregnancy, vertical transmission, epidemiological surveillance, Croatia

## Abstract

Four infants potentially exposed to syphilis infection in utero, meeting World Health Organization surveillance criteria of congenital syphilis (CS), were diagnosed in Croatia between September 2020 and January 2024. We conducted a retrospective analysis of epidemiological, clinical and laboratory data of these cases to assess compliance with surveillance case definitions. As only one confirmed CS case has been reported in Croatia in over 2 decades, these reports signal an increased risk of syphilis vertical transmission and warrant strengthening antenatal screening.

Syphilis can be transmitted sexually, through blood products or vertically during pregnancy [1]. Over half of pregnancies in women with active syphilis result in adverse outcomes, including stillbirth, early neonatal death or neonatal infection [2]. Except for one case reported in 2009, congenital syphilis (CS) has not been registered in Croatia for over 2 decades. Between September 2022 and September 2024, four cases of CS have been reported to the Croatian Institute of Public Health (CIPH), alerting a considerable increase in the risk of vertical transmission [3]. Here, we present national surveillance data and retrospective clinical and laboratory findings of the four CS cases diagnosed in Croatia in the period from September 2020 to January 2024 to explore possible increased risk of vertical transmission of syphilis in Croatia.

## Congenital syphilis surveillance in Croatia

In Croatia, syphilis and CS are mandatory notifiable diseases since 1958 and current surveillance is based on the European Union (EU) case definition [4,5]. Compared to an average of 34 cases during 2008–19, the number of reported syphilis cases overall increased from 29 in 2020 to 56 in 2022, then decreased to 37 in 2023. Of these, 5, 7 and 3 of the cases were among women, respectively [6]. This is of particular concern given that, in Croatia, syphilis testing has not been routinely offered during prenatal care in more than 2 decades but is typically conducted on a case-by-case basis, prompted by specific epidemiological or medical considerations [7]. 

## Epidemiological, laboratory and clinical findings

We conducted a retrospective analysis of four infants managed as suspected CS cases in clinical settings in Croatia between September 2020 and January 2024 and reported to the CIPH between September 2022 to September 2024. Clinical and epidemiological data were collected for each case through hospital medical records, the national electronic information surveillance system and personal communications with physicians. These cases were reviewed according to the EU and World Health Organization (WHO) surveillance CS case definitions [2,4], and United States Centers for Disease Control and Prevention (US CDC) Sexually Transmitted Infections Treatment Guidelines (2021) clinical criteria for CS [8]. The EU criteria of case definition for a confirmed case requires a specific combination of laboratory tests in the child that may not be routinely performed in clinical settings. The WHO surveillance case definition allows for reporting of CS based on the positive syphilis serology in the mother or when syphilis treatment in the mother was inadequate. The US CDC criteria are intended to guide clinical practice, allowing for a combination of clinical, radiological or epidemiological evidence to direct a CS case workup. The WHO surveillance case definition is intended to be more sensitive to allow better ascertainment of vertical transmission, as many countries do not have all types of tests available for appropriate laboratory confirmation of CS. Epidemiological and clinical data of cases are shown in the Table.

**Table:** Clinical and epidemiological characteristics of infants clinically diagnosed as congenital syphilis and screening/treatment of parents, Croatia, September 2020–January 2024 (n = 4)

Characteristics	Patient 1	Patient 2	Patient 3	Patient 4
Sex of infant	Female	Male	Female	Female
Year of birth	2020	2022	2023	2024
Maternal care				
Syphilis screening test results of the mother and week of gestation	RPR + (1:16); TPHA + (10,240) Week 10	RPR + ; TPHA + (1,280); anti-TP IgM + , IgG + Week 31	RPR + (1:4); TPHA + (2,560); anti-TP + (22.7) Week 17	RPR − ; TPHA + (320); anti-TP IgM + , IgG + Week 32
Mother’s treatment (period during pregnancy or after delivery; diagnosed stage of syphilis)	Penicillin G benzathine 2.4 million units IM once weekly, 3 doses (first and early second trimester; latent syphilis of unknown duration – treated as late latent syphilis)	Penicillin G benzathine 2.4 million units IM once weekly, 3 doses (after delivery; latent syphilis of unknown duration – treated as late latent syphilis)	Penicillin G benzathine 2.4 million units IM once weekly, 3 doses (second trimester; latent syphilis of unknown duration – treated as late latent syphilis)	Penicillin G benzathine 2.4 million units IM once weekly, 3 doses (weeks 35–37; latent syphilis of unknown duration – treated as late latent syphilis)
Repeated maternal screening and week of gestation	RPR − ; TPHA + (320) Week 31	RPR + (1:32); TPHA + (5,120); anti-TP + (27.13) 4 weeks after delivery, at the same time as the infant's initial screening	RPR + (1:4); TPHA + (1,280) Week 37	RPR − ; TPHA + (80); Anti-TP borderline Week 37
Clinical and laboratory findings in infants				
Age at disease onset/hospitalisation for CS (symptoms status)	1 day (asymptomatic)	7 weeks (symptomatic)	1 day (asymptomatic)	9 days (asymptomatic)
Presumed diagnosis at admission	Neonatal sepsis/CS	Neonatal sepsis/CS	Neonatal sepsis/CS	Healthy, possible CS
Fever	No	Yes	No	No
Skin lesions	No	Yes, multiple, including palms and soles	No	No
PCT (µg/L)	2.9	82.3	NA	0.1
CRP (mg/L)	0.7	209.5	11.2	0.9
White blood cell count (x10^9^/L)	27.9	8.8	30.7	12.6
Red blood cell count (x10^12^/L)	5.0	2.6	6.1	5.24
Haemoglobin (g/L)	168	78	210	183
Platelet count (x10^9^/L)	246	58	216	502
AST (U/L)	65	26	42	56
ALT (U/L)	17	13	18	36
GGT (U/L)	NA	186	128	NA
Bilirubin (µmol/L)	105; indirect 101, direct 4	54; indirect 28, direct 26	79; indirect 73, direct 6	127; indirect 117, direct 10
LDH (U/L)	663	220	473	NA
Coagulation tests	NA	Normal	NA	NA
CSF findings	NA	Pleocytosis	NA	NA
CSF culture	NA	Sterile	NA	NA
Cell count (cells/μL)	NA	29	NA	NA
TPHA (CSF)	NA	Negative	NA	NA
Blood culture	NA	Sterile	Sterile	NA
Urine culture	NA	Sterile	NA	NA
Chest X-ray	NA	Bilateral pneumonia	NA	NA
Abdominal ultrasound	Normal	Hepatosplenomegaly	Normal	NA
Cranial ultrasound	Periventricular echogenicity	Normal	NA	Normal
Screening, treatment and follow-up in infants				
TPHA (age at first screening)	Positive, 160 (1st week)	Positive, 320 (4th week)	Positive, 640 (1st week)	Positive, 80 (9th day)
		Positive, 320 (7th week)		
RPR (age at first screening)	Negative (1st week)	Negative (4th week)	Positive, 1:4 (1st week)	Negative (9th day)
		Positive, 1:16 (7th week)		
FTA-ABS (age at first screening)	IgM negative; IgG positive, 80 (1st week)	NA	NA	NA
Anti-TP (age at first screening)	NA	Positive, 22.91 (4th week)	Positive, 23.9 (1st week)	Borderline (9th day)
		Positive, 22.02 (7th week)		
Treatment	Ampicillin IV 14 days; Gentamycin IV 10 days; Azithromycin PO 5 days	Ampicillin and cefotaxime on day 1 of hospitalisation, then penicillin G IV 10 days followed by single dose of benzathine penicillin G IM ^a^	Ampicillin and gentamycin IV 7 days, then crystalline penicillin G IV 10 days followed by single dose benzathine penicillin G IM ^a^	Single dose of benzathine penicillin G IM ^a^
Outcome	Favourable	Favourable	Favourable	Favourable
TPHA (age at follow-up after hospital discharge)	Positive, 640 (5 weeks)	Positive, 320 (11 weeks)	Positive, 160 (8 weeks)	Negative (8 weeks)
	Positive, 160 (12 weeks)			
RPR (age at follow-up after hospital discharge)	Negative (5 weeks)	Positive, 1:16 (11 weeks)	Negative (8 weeks)	NA
	Negative (12 weeks)			
FTA-ABS (age at follow-up after hospital discharge)	IgM negative; IgG positive 160 (5 weeks)	NA	NA	NA
	IgM negative; IgG positive 20 (12 weeks)			
Anti-TP (age at follow-up after hospital discharge)	NA	Positive, 21.83 (11 weeks)	NA	NA
HIV testing (mother)	Performed, negative	Performed, negative	NA	Performed, negative
HIV testing (infant)	NA	Performed, negative	NA	NA
Further follow-up screening of infant	RPR and FTA-ABS negative at 16 weeks of age	Not performed, did not attend appointment	Not performed, did not attend appointment	Not performed
Syphilis testing, diagnosis and treatment of infant’s father	Tested and laboratory confirmed positive during pregnancy, treated	Tested, negative, not treated	Unknown	Tested, negative, not treated
Case definitions and classifications				
Case classification (EU) [4]	Probable	Probable	Probable	Possible - NA
Diagnosis category according to treatment guidelines (US CDC) [8]	Possible	Confirmed proven/highly probable	Possible	Possible
WHO surveillance case definition [2]	Congenital syphilis	Congenital syphilis	Congenital syphilis	Congenital syphilis

Anti-TP: anti-*Treponema pallidum* test, chemiluminescence immunoassay (index); ALT: alanine transaminase; AST: aspartate transferase; CS: congenital syphilis; CRP: C-reactive protein; CSF: cerebrospinal fluid; EU: European Union; FTA-ABS: fluorescent treponemal antibody absorption (index); GGT: gamma-glutamyl transferase; HIV: human immunodeficiency virus; IM: intramuscular; IV: intravenous; LDH: lactate dehydrogenase; NA: not analysed or applicable; PCT: procalcitonin; PO: peroral; RPR: rapid plasma reagin test (titre); TPHA: *Treponema pallidum* hemagglutination assay (titre); US CDC: United States Centers for Disease Control and Prevention; WHO: World Health Organization.

^a^ Single dose of 50,000 U/kg benzathine penicillin G IM.

Reference values (normal range) for laboratory tests are as follows: ALT (U/L): 11–46; AST (U/L): 26–75; bilirubin direct (µmol/L): < 3; bilirubin indirect (µmol/L): < 15; bilirubin total (µmol/L): < 20; cell count - cerebrospinal fluid (cells/μL): < 20; CRP: 0.1–4.1; GGT (U/L): 15–132; haemoglobin (g/L): 136–200; LDH (U/L) 150–360; PCT: < 0.5; platelet count (x10^9^/L): 150–450; red blood cell count (x10^12^/L): 3.9–5.5; white blood cell count (x10^9^/L): 6.2–17.8.

Among reported cases, three neonates were full-term, while Patient 2 was born late preterm at 36 weeks and 1 day. All mothers were longtime Croatian residents, except for Patient 3, whose parents had returned from another EU country 2 years prior to the pregnancy. 

Patient 2 developed signs of infection in the seventh week of age, starting with a papulosquamous rash followed by fever 2 days later and is the only confirmed case, according to the US CDC treatment guidelines [8]. The mother was tested 5 weeks before delivery, but the positive results were only identified retrospectively at the infant's first paediatric visit at 1 month of age. One week before hospital admission, the infant was evaluated and screened as a possible CS case. Soon after, the child presented with symptoms at another hospital where, due to the presumed diagnosis of neonatal sepsis and unawareness of maternal history of syphilis, treatment was initiated with ampicillin and cefotaxime. On the second day, the infant was transferred to a hospital where they had previously been evaluated, therapy was switched to penicillin, and the child recovered completely. Based on clinical and available diagnostic findings, the diagnosis of CS was confirmed.

Patient 1's mother was diagnosed with syphilis via positive serology early in pregnancy during her regular first visit to a gynaecologist, when she received treatment. After delivery, the newborn was asymptomatic, and serology findings suggested transplacentally-acquired treponemal antibodies. Due to elevated inflammatory markers, empirical antibiotic treatment for neonatal sepsis was conducted, but the infant did not receive penicillin therapy. Clinical improvement despite inadequate antibiotic therapy along with negative results of follow-up syphilis screening tests do not support the diagnosis of CS as per EU surveillance case definition and CDC treatment guidelines while meeting the WHO surveillance case definition of CS.

Mother of Patient 3 diagnosed with syphilis during the first trimester, when she was tested owing to a previous spontaneous abortion several years before the present pregnancy; she received treatment during second trimester of pregnancy. Although the newborn child appeared healthy after birth, a 7-day antibiotic treatment regimen was administered for possible early neonatal sepsis given a high leukocyte count. The child had positive treponemal tests but with rapid plasma reagin (RPR) values equal to the mother. Despite likely passive acquisition of treponemal antibodies, she received additional treatment for CS and was discharged soon thereafter.

Normalisation of laboratory tests during non-penicillin therapy in both Patients 1 and 3, along with appropriate maternal treatment, insufficient diagnostic work-up for neonatal sepsis and clinical presentation, suggest that these infants were more likely to have had neonatal infections or sepsis caused by undetected bacteria than CS. 

For the most recent case, Patient 4, the mother had a positive treponemal test and a negative RPR detected 6 weeks before delivery, indicating possible late latent syphilis or a very early infection. She completed treatment 3 weeks before delivery. Since the mother's treatment was not timely, the newborn was considered as having been potentially exposed to syphilis and received a single dose of penicillin. The infant evolved favourably, with negative treponemal tests 2 months after hospital discharge. A CS diagnosis was not confirmed.

Syphilis screening for the mothers of Patients 2 and 4 was performed because they were under 18 years of age with pregnancies detected late, having their first gynaecological appointment during the last trimester. Their partners were also screened and tested negative. The father of Patient 1 was diagnosed with syphilis during his partner’s pregnancy and received appropriate treatment. 

## Discussion

After retrospective analysis, none of the patients can be classified as confirmed case according to the EU CS case definition for surveillance [4], but all four meet WHO surveillance case definition of CS [2]. Patient 2 had a typical clinical manifestation of neonatal CS, a positive epidemiological link, positive treponemal and non-treponemal tests and, as such, meets the US CDC treatment guidelines criteria for a confirmed CS case [8-10]. However, given the lack of availability of certain diagnostic tools (such as dark field microscopy, direct fluorescent antibody staining for *Treponema pallidum* (DFA-TP) demonstration in skin lesions, *T. pallidum*-specific IgM), with only chemiluminescence immunoassays (CLIA) and *T. pallidum* hemagglutination assays (TPHA) performed, this patient might be considered a probable case, along with Patients 1 and 3, while Patient 4 does not meet the EU surveillance case definition criteria [4]. Notably, there is discrepancy between the US CDC diagnostic categories for CS used by clinicians and surveillance case definitions [4,8].

The reported cases highlight several concerning issues regarding CS in Croatia. To facilitate early detection and timely treatment of pregnant women and to prevent CS, it is strongly recommended to raise awareness of health professionals about the upsurge of syphilis and CS in the EU and to re-introduce universal syphilis screening for pregnant women at their first antenatal care visit [11]. Repeat testing should be provided for those women and their partners engaging in high-risk behaviours [12]. Of particular importance is to improve availability of laboratory diagnostics for confirmatory testing for CS.

In the European Union/European Economic Area (EU/EEA) in 2022, 69 cases of CS were reported from 14 of 25 countries submitting data to the European Centre for Disease Prevention and Control (ECDC). This was an increase of 25% compared to the number of reported cases in 2021 (55 cases from 11/24 countries). Similar upsurges were also noted with other sexually transmitted infections (STIs) [13-15]. Furthermore, a deeper understanding of the syphilis epidemic in Croatia is necessary. Finally, it should be noted that CS can mimic neonatal sepsis of other aetiologies, necessitating a high index of clinical suspicion for accurate diagnosis [16]. Clinicians should be aware of the possibility of congenital syphilis, even when risk factors are unknown, to ensure proper treatment and prevent sequelae.

## Conclusion

Considering recent increases in syphilis and other sexually transmitted infections in the EU/EEA among the heterosexual population, including women of reproductive age, it is important to revise the EU case definition and allow probable cases to be reported in order to better ascertain the risk of vertical transmission of syphilis. Moreover, the findings highlight the necessity of reintroduction of universal syphilis testing in the first trimester of pregnancy in Croatia.
